# Compared the Microbiota Profiles between Samples from Bronchoalveolar Lavage and Endotracheal Aspirates in Severe Pneumonia: A Real-World Experience

**DOI:** 10.3390/jcm11020327

**Published:** 2022-01-10

**Authors:** Yeong-Nan Cheng, Wei-Chih Huang, Chen-Yu Wang, Pin-Kuei Fu

**Affiliations:** 1 Institute of Bioinformatics and Systems Biology, College of Biological Science and Technology, National Yang Ming Chiao Tung University, Hsinchu 300, Taiwan; tom.cheng0911@gmail.com (Y.-N.C.); loveariddle.bi96g@g2.nctu.edu.tw (W.-C.H.); 2 Department of Biological Science and Technology, College of Biological Science and Technology, National Yang Ming Chiao Tung University, Hsinchu 300, Taiwan; 3 Department of Nursing, Hungkuang University, Taichung 43302, Taiwan; cheuyu@vghtc.gov.tw; 4 Department of Critical Care Medicine, Taichung Veterans General Hospital, Taichung 40705, Taiwan; 5 College of Human Science and Social Innovation, Hungkuang University, Taichung 433304, Taiwan; 6 Ph.D. Program in Translational Medicine, National Chung Hsing University, Taichung 402010, Taiwan

**Keywords:** bronchoalveolar lavage, endotracheal aspirate, microbiota profiles, pneumonia, respiratory failure

## Abstract

Lower respiratory tract sampling from endotracheal aspirate (EA) and bronchoalveolar lavage (BAL) are both common methods to identify pathogens in severe pneumonia. However, the difference between these two methods in microbiota profiles remains unclear. We compared the microbiota profiles of pairwise EA and BAL samples in ICU patients with respiratory failure due to severe pneumonia. We prospectively enrolled 50 ICU patients with new onset of pneumonia requiring mechanical ventilation. EA and BAL were performed on the first ICU day, and samples were analyzed for microbial community composition via 16S rRNA metagenomic sequencing. Pathogens were identified in culture medium from BAL samples in 21 (42%) out of 50 patients. No difference was observed in the antibiotic prescription pattern, ICU mortality, or hospital mortality between BAL-positive and BAL-negative patients. The microbiota profiles in the EA and BAL samples are similar with respect to diversity, microbial composition, and microbial community correlations. The antibiotic treatment regimen was rarely changed based on the BAL findings. The samples from BAL did not provide more information than EA in the microbiota profiles. We suggest that EA is more useful than BAL for microbiome identification in mechanically ventilated patients.

## 1. Introduction

According to the World Health Organization statistics, pneumonia was the fourth-leading cause of death in 2016, causing 3.0 million deaths worldwide [1,2]. Conventional culture-based sputum analysis remains the primary tool for the diagnosis and management of pneumonia [3]. The methods used to collect respiratory specimens include invasive methods such as bronchoalveolar lavage (BAL) and noninvasive methods such as endotracheal aspiration (EA) [4,5]. Previous trials observed no significant differences between these methods with respect to 28-day mortality, overall mortality, length of ICU stay, duration of mechanical ventilation, or changes in antibiotic treatment [6,7,8]. Some reports revealed that quantitative culture results from BAL may be beneficial in guiding the discontinuation of antibiotics to shorten their administration time and to improve microbiologic outcomes [7,9,10]. However, these studies comparing BAL and EA with respect to pneumonia diagnosis and treatment do not provide strong recommendations, have low-quality evidence, and cite older references published in 1999–2013 [1,7,11,12].

In recent years, 16S rRNA metagenomic sequencing has been used to analyze microbiota profiles in EA and BAL samples [13,14]. This novel, culture-independent technique can identify diverse communities of bacteria previously undetected by culture-based approaches [15]. This technique has been used to evaluate the association of chronic obstructive pulmonary disease (COPD) exacerbations with lung microbiome dysbiosis [16] and with the severity of bronchiectasis [13,17] and cystic fibrosis [18]. However, the microbiota profile has not been systematically compared between the samples from EA and BAL in severe pneumonia with acute respiratory failure.

The primary aim of the current study is to compare the microbiota profile by using 16S rRNA metagenomic sequencing methods in EA and BAL samples. In addition, we also analyzed the antibiotic prescription patterns between patients with negative and positive BAL cultures. We compared the microbiota profiles between EA and BAL samples by using next-generation sequencing (NGS) methods to determine the relative microbe abundance, diversity, and correlation networks.

## 2. Materials and Methods

### 2.1. Study Design

This prospective cohort study was conducted at the medical intensive care unit (ICU) of Taichung Veterans General Hospital (TCVGH), a medical center in Central Taiwan, from September 2017 to May 2018. The study protocol was fully reviewed and approved by the Institutional Review Board of TCVGH (IRB number, SE17214B; date of approval, 29 August 2017) (ClinicalTrials.gov Identifier, NCT03379779).

### 2.2. Patients, ICU Setting, and Severity Scores

The 41-bed medical ICU in which this study was conducted admitted adult patients, the majority of whom were diagnosed with pneumonia-related sepsis, septic shock, or acute respiratory failure. The study cohort included adults aged >40 years with a diagnosis of new onset of pneumonia within 48 h accompanied by acute respiratory failure requiring support with mechanical ventilation, with bronchoalveolar lavage (BAL) conducted. EA and BAL samples were obtained from enrolled patients at the same time. We excluded patients diagnosed with active pulmonary tuberculosis and those treated with antibiotics for more than 7 days for any reason within two weeks of developing the new episode of pneumonia-related acute respiratory failure. All patients were evaluated and asked to give written consent for study participation on the day of admission. The indications and risks associated with BAL were described by the pulmonary specialist. Some of the enrolled patients had informed consent forms signed by their surrogates. All enrolled patients were treated following the standard ICU protocols, including the lower tidal volume ventilator strategy for acute respiratory distress syndrome (ARDS), nutritional support, antibiotic treatment, and hemodialysis, according to the recent guidelines or consensus [1,4,19,20,21,22,23]. Bronchoscopic examination and BAL were performed in our ICU by three full-time intensivists who are also registered pulmonologists. Nutrition evaluation was performed in the ICU by a registered dietitian according to the modified NUTRIC (mNUTRIC) score [24] and severity scores, including the Acute Physiology and Chronic Health Evaluation (APACHE) II and Sequential Organ Failure Assessment (SOFA). Scores were evaluated by an advanced practice registered nurse as previously described [4,22].

### 2.3. Data Collection, Sample Collection, and DNA Extraction

Data collected from the case report form included age; sex; body mass index (BMI); and clinical outcomes, including the severity of the illness (APACHE II and SOFA scores on ICU day 1); major comorbidities and the Charlson Comorbidity Index (CCI); the number of ventilator, hospitalization, and ICU days; and mortality in the ICU or hospital. In this cohort, enrolled lower respiratory tract specimens were routinely collected by EA and selectively collected by BAL on the first ICU day. Samples from EA and BAL were sent to the microbiology culture for bacterial identification and the microbiology laboratory at TCVGH for DNA extraction. All patients enrolled into this study obtained EA for bacterial identification on the third ICU day to follow the change of pathogens after treatment. 

DNA was extracted directly from EA and BAL samples using the QIAamp DNA Blood Mini Kit (Qiagen, Hilden, Germany). Each sample was transferred to a 1.5-mL microcentrifuge tube and centrifuged at 14,000 rpm for 2 min to pellet the bacteria. Bacterial pellets were suspended in 180 µL of the appropriate enzyme solution and incubated for at least 30 min at 37 °C. In addition, 20-µL proteinase K and 200-µL Buffer AL were added to the sample and mixed by vortexing. Each suspension was incubated at 56 °C for 30 min and then for a further 15 min at 95 °C. The 1.5-mL microcentrifuge tube was briefly centrifuged to spin the suspension. DNA was then extracted following the manufacturer’s protocols. The extracted DNA was then eluted with 30-µL Buffer AE and centrifuged at 8000 rpm for 1 min. Final concentrations were measured using NanoPhotometer Pearl (Implen GmbH, Munich, Germany), and the samples were stored at −20 °C until further analysis.

### 2.4. Library Construction and 16S Ribosomal RNA Sequencing

For two-step PCR amplification, adapter sequences were added to the V4 hypervariable regions, providing ample information for the taxonomic classification of microbial communities according to the human microbiome studies in the Human Microbiome Project [25]. To provide a more balanced base composition through the entire duration of the sequencing run, we added a “heterogeneity spacer” with a length of 1–3 base pairs to the first PCR primers [26,27]. After each PCR step, amplicons were purified using the AMPure XP PCR Purification Kit (Beckman Coulter Genomics, Danvers, MA, USA) and quantified using the Qubit dsDNA HS Assay Kit (Qubit, Thermo Fisher Scientific, Waltham, MA, USA) and a Qubit 3.0 Fluorometer (Qubit, Thermo Fisher Scientific), according to the respective manufacturers’ instructions, using the Library Quantification Kit for Illumina (Kapa Biosciences, Woburn, MA, USA). Finally, the library was mixed with 20% of the Illumina internal sequencing PhiX control library (v3) (Illumina, San Diego, CA, USA) with a cluster density of 800–950 K/mm^2^ in 250 paired-end MiSeq runs. After the sequencing program was completed, image analysis, base calling, and data quality assessments were performed using the MiSeq instrument.

### 2.5. Filtering 16S rRNA Sequencing Data for Quality

Sequencing reads from different samples were identified and separated using a specific barcode at the 5′ end of the sequence (two mismatches allowed). The FASTX-Toolkit [28] was employed to process raw read data files. Three steps were followed to ensure sequence quality processing:

(i) The command “fastq_quality_filter −Q 33 −q 20 −p 70”. “−q 20” designated 20 as the minimum quality score. “−p 70” designated the minimum percentage of the bases required for “−q” quality to be ≥70%.
(ii) The command “fastq_quality_trimmer −t 20 −l 100 −Q 33”. “−t 20” designated that bases with lower qualities (Q < 20) would be trimmed (checking from the end of the sequence). “−l 100” indicated that the minimum acceptable sequence length was 100 after trimming.
(iii) Sequences were retained if both forward and reverse sequencing reads passed the first and second steps.

### 2.6. Taxonomy Assignment Based on Bacterial 16S rRNA Sequences

After sequencing data preprocessing, QIIME was used for performing the microbiome analysis [29]. To assign taxonomy, a collection of 16S rRNA sequences was retrieved from the Greengenes database (version13_8) [30]. These sequences were extracted with V4 forward and reverse primers. The UCLUST tool then was used to create representative sequence clusters with similarities ≥97% [31]. Bowtie2 was used to align sequencing reads against V4 sequence clusters [32]. A 97% similarity standard was applied to the V4 sequence clusters.

### 2.7. Computational and Statistical Analysis

After taxonomy assignment, an OTU table was generated. Taxa of the same level were agglomerated at the phylum, class, order, family, and genus levels via the Greengenes database. To explore the association between the bacterial community and factors related to individuals, the alpha diversity analysis (Shannon index and Chao index) was performed, and the biodiversity between groups was compared. To investigate the relationships between specific genera with a presence rate ≥50%, Spearman’s rank correlation coefficients were calculated to construct a correlation network.

Data were analyzed using R software (The R Project for Statistical Computing, Vienna, Austria). Categorical variables are presented as the frequency and percentage and were analyzed using the chi-square test to determine significance. For nonparametric distribution data, differences between groups were assessed using the Mann–Whitney *U* test or Wilcoxon signed-ranks test; results were presented as the median and interquartile range (IQR). Cox regression analysis was used to assess the factors associated with mortality. All tests were performed with two-sided tests, where *p* < 0.05 was considered statistically significant.

## 3. Results

### 3.1. Cohort Demographic Data and Antibiotic Prescription Pattern

**Figure 1:**
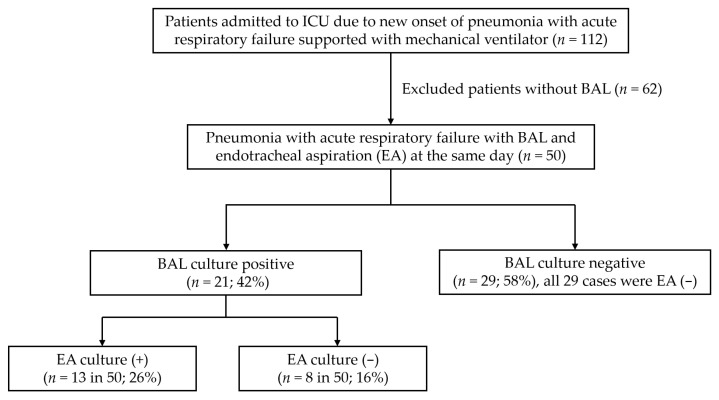
Study flow chart. EA, endotracheal aspiration; BAL, bronchoalveolar lavage.

Table 1 shows the demographic characteristics, severity scores, comorbidities, clinical outcomes, and antibiotic prescription patterns in this cohort. A total of 50 patients (17 women, 33 men) were enrolled in this study. The APACHE II, SOFA, and mNUTRIC scores indicated that these patients had severe illness and nutrition risk. The mortality rates were 26.0% in the ICU and 36.0% in the hospital. The most common comorbidities were cerebral vascular disease, cardiovascular disease, chronic obstructive lung disease, malignancy, and diabetes mellitus. The pathogens were identified in a culture medium from BAL samples in 21 (42%) patients and from both BAL and EA samples in 13 (26%) out of 50 patients (Figure 1). Antibiotics at the first ICU day were prescribed to 78% for anti-*Pseudomonas* coverage and 6% for both anti-*Pseudomonas* and anti-*MRSA* coverage. The antibiotic prescribed was changed based on the BAL culture report at the third ICU day in only 18% (*n* = 9) of the cohort; most of this change was a de-escalation of anti-Pseudomonas antibiotics (77.8%; *n* = 7) (Table 1).

### 3.2. Subgroup Analysis of Clinical Outcomes and Antibiotic Prescription Pattern According to BAL Culture Results

Comparisons of the demographic characteristics, severity scores, comorbidities, clinical outcomes, and antibiotic prescription patterns between BAL-positive and BAL-negative patients are shown in Table 2. No significant difference was observed between BAL-positive and -negative patients with respect to the APACHEII score, SOFA score, CCI, comorbidities, or clinical outcomes. The antibiotics prescribed on day 1 and after BAL culture reporting also did not differ between the two groups. Most patients were treated with anti-Pseudomonas antibiotics initially, and only 20% of this cohort had their antibiotics de-escalated based on the BAL culture report.

### 3.3. The EA Microbiota Resembles BAL Microbiota

**Figure 2:**
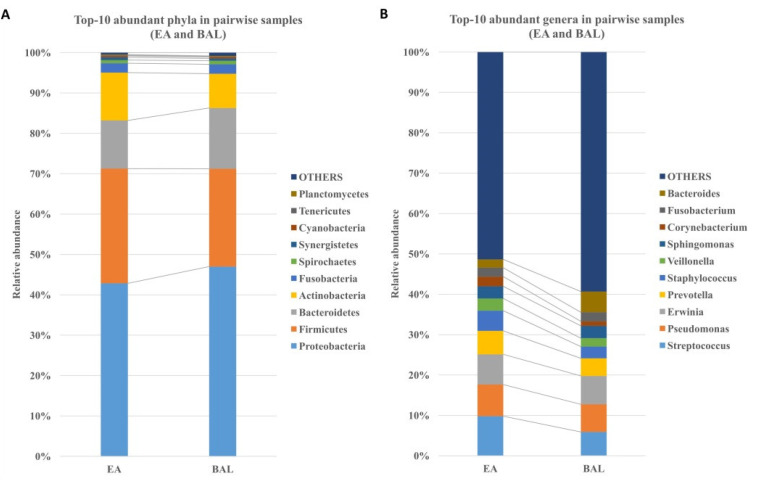
Relative abundance of the major microbiota in each pairwise EA and BAL samples. (**A**) Top 10 abundant phyla. (**B**) Top 10 abundant genera.

The 10 most abundant phyla and genera were similar between the EA and BAL samples (Figure 2). The Spearman’s rank correlation coefficient (rho) between pairwise EA and BAL samples was calculated using the relative abundance at the genus level for each subject and showed that the microbial compositions’ positive correlation was high (rho ≥ 0.7) in 32% of the patients (16/50) and moderate (rho ≥ 0.4) in 76% of the patients (38/50). In addition, each subject’s EA and BAL microbiota were more similar to each other than to those of other subjects. The NGS analysis of the 20 most common ICU pathogens showed no significant differences between the EA and BAL specimens (Figure 2). These results suggested a high degree of consistency in culture-independent detection between the EA and BAL specimens.

### 3.4. Similar Microbial Diversity in EA and BAL Samples

**Figure 3:**
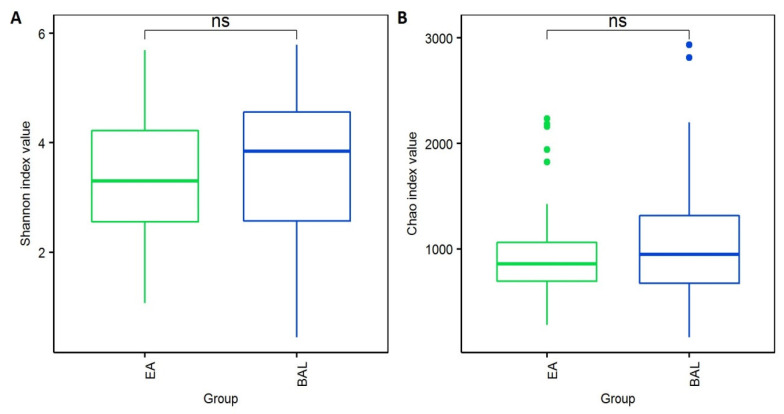
Comparison of the microbial diversity between the EA and BAL samples according to (**A**) the Shannon index and by (**B**) the Chao index. ns: no significant difference.

To summarize the diversity of the microbiota in different specimens, the Shannon (species evenness) and Chao (species richness) indices were determined (Figure 3). Neither index differed significantly between the EA and BAL samples (*p* > 0.05), indicating that the microbial compositions of these two types of specimens were similar.

### 3.5. Consistent Microbial Relationships between EA and BAL Samples

**Figure 4:**
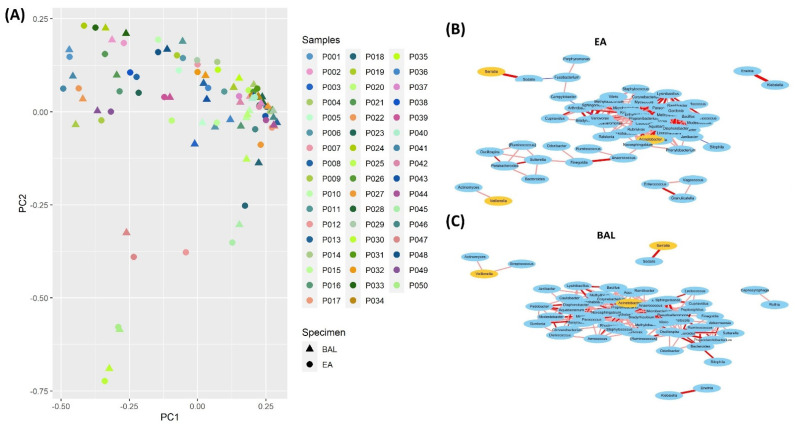
Microbial composition in the EA samples resembled that in the BAL samples for most patients. (**A**) Results of the Principal Coordinates Analysis (PCoA) of the 50 patients with CAP pairs using Bray-Curtis distance. (**B**) Counterbalances between the microbiota in the EA samples. (**C**) Counterbalances between the microbiota in the BAL samples.

We compared the composition of microbial communities between the EA and BAL samples. Spearman’s rank correlation coefficients were calculated for 79 genera, each of which had a presence ≥50% in both the EA and BAL samples. The correlation between microbial communities was the same in the EA (Supplementary Figure S1A) and BAL samples (Supplementary Figure S1B). This result indicates that correlations between two genera were highly similar in the EA and BAL samples, but negative correlations of some microbiota were more apparent in the EA samples. In addition, correlations between the microbial communities were highly consistent (rho, 0.721) between the EA and BAL samples (Supplementary Figure S1C). In Figure 4A, it showed high similarities of the microbial compositions between the EA and BAL samples for the CAP patients by the Principal Coordinates Analysis (PCoA) using Bray-Curtis distance. The distance between the samples indicated the similarity of the microbial community in the sample. The closer the samples, the higher the similarities of the microbial compositions. For most patients, the distance between the EA and BAL samples collected from the same patient was short. Comparison of the microbial correlation network of the EA and BAL samples revealed that counterbalances between microbiota were more apparent in the EA samples (Figure 4B,C). Therefore, the relationships of the microbial communities were consistent between the EA and BAL samples.

## 4. Discussion

The current study compares the composition of microbial communities in endotracheal aspirate (EA) and bronchoalveolar lavage (BAL) samples in a cohort of mechanically ventilated pneumonia patients. We observed no significant differences in antibiotic prescription patterns between patients with and without positive BAL cultures. We also observed a significant similarity in the microbiota present EA and BAL samples, including the relative abundance, diversity, and correlation network of the communities. We conclude that lower respiratory tract sampling by EA may be a better choice than BAL, because it is both more convenient technically and provides more information regarding the microorganisms.

We report three major findings in this study. First, no significant difference was observed in the antibiotic prescription patterns between patients with positive and negative culture results from bronchoalveolar lavage (BAL) samples. Although the positive rate of the cultures was higher for the BAL (42%) than for the endotracheal aspiration (26%) samples, less than 20% of these patients were prescribed a change in antibiotic prescription on day 3 based on the BAL results. In fact, several previous large clinical trials showed that invasive respiratory sampling such as BAL did not lead to a significant difference in the 28-day mortality, overall mortality, length of ICU stay, mechanical ventilation days, or antibiotic changes [6,7,8]. In a prospective study comparing the effectiveness of routine endotracheal aspirate (EA) cultures compared to BAL cultures for diagnosing ventilator-associated pneumonia, Yagmurdur et al. observed a high correlation between the EA and BAL samples with respect to the microorganism antibiotic resistance patterns [32]. In our cohort, 84% of the patients were empirically prescribed anti-*Pseudomonas* antibiotics initially for severe pneumonia with septic shock, and 80% of them continued this treatment even after the BAL bacterial cultures were negative. In addition, advanced antibiotics coverage in aged patients with multiple comorbidities was associated with a low frequency of the positive culture rate of *Streptococcus pneumonia* in the cohort. This finding may be attributed to the concern of factors that increase the risk of recurrent admission and infection by multiple drug-resistant pathogens, such as advanced patient age, high disease severity, high nutritional risk, and multiple comorbidities [33]. Moreover, mechanical ventilation in patients with severe pneumonia was associated with the dysbiosis of microbial communities in the low respiratory tract that are more profound in patients who develop VAP [34].

Secondly, we observed a high correlation between the microbial community composition of the EA and BAL samples, as determined by next-generation sequencing [33]. Previous studies of BAL samples report that *Prevotella*, *Bacteroides*, and *Veillonella* were common in the bacterial flora of the lower respiratory tracts of healthy subjects, as well as *Fusobacterium*, *Streptococcus*, and *Pseudomonas* [35,36,37]. Kazuhiro et al. reported that the dominant phylotypes from the BAL analysis in patients with community-acquired pneumonia (CAP), healthcare-associated pneumonia (HCAP), and hospital-acquired pneumonia (HAP) were oral *streptococci* and *anaerobes* [38,39]. Another previous study comparing the culture-dependent and NGS methods for identifying methicillin-resistant *Staphylococcus aureus* (MRSA) pneumonia reported that the culture method may over-detect MRSA [39]. In our study, we found the 10 most abundant genera were identified as *Streptococcus*, *Pseudomonas*, *Erwinia*, *Prevotella*, *Staphylococcus*, *Veillonella*, *Sphingomonas*, *Corynebacterium*, *Fusobacterium*, and *Bacteroides* in both the EA and BAL samples. A high correlation of the microbial existed between the EA and BAL samples.

Finally, for patients on mechanical ventilation, we suggest that lower respiratory tract sampling by EA may be a better choice than BAL, because it is both technically more convenient and provides more information regarding the microorganisms. Our results revealed that the microbiota profiles in EA and BAL samples are similar with respect to diversity, microbial composition, and microbial community correlations. Recently, the NGS method has been applied very commonly for evaluating microbiota profiles in chronic lung diseases, such as chronic obstructive pulmonary disease (COPD), bronchial asthma, and interstitial lung disease [40,41,42]. Many studies have used EA to investigate microbiomes in COPD because of its technical ease and standardized processing procedure [40]. Although a previous study suggested that EA and BAL samples provide different representations of the lung microbiome [36], however, we found high similarities in the microbiota profiles between EA and BAL in pneumonia patients. We suggested that a sample analysis is more clinically useful for EA than for BAL, providing a noninvasive, technically simple procedure for identifying the lung microbiome in mechanically ventilated patients.

The current study has several limitations. First, this research is limited by the small sample size. Second, EA and BAL data from healthy controls are not included because of ethical concerns regarding the recruitment of normal subjects for EA and BAL. Third, the EA and BAL samples used in this study could have been contaminated with upper airway or residual oral bacteria. Fourth, patients who smoked or had malignancies or comorbidities involving other organs or systemic disorders were not excluded from this study. Thus, the effect of underlying disease on the microbiome could be an issue. Fifth, most of the enrolled patients were also treated with systemic steroids for septic shock; therefore, the composition of the microbiome may have been influenced by steroid use.

## 5. Conclusions

The antibiotic treatment regimen rarely changed based on the BAL findings. The samples from bronchoalveolar lavage (BAL) did not provide more information than endotracheal aspirates (EA) in the microbiota profiles by using the culture-independent 16S rRNA metagenomic sequencing method. We suggest that EA is a more convenient sample than BAL for microbiome 16S rRNA metagenomic sequencing in mechanically ventilated patients to provide equivalent information and less invasive.

## Figures and Tables

**Table 1:** Demographic characteristics, severity scores, comorbidities, and clinical outcomes of all pneumonia patients undergoing bronchoalveolar lavage (BAL) in the intensive care unit (*n* = 50).

Characteristics (*n* = 50)	Median IQR (*n*, %)	
Age	75.4	66–84.5
Gender (*n*, Male %)	33	66.0%
Body mass index	22.5	19.5–25.8
APACHEII	29	24.8–34
SOFA score	7.5	5.8–10
mNURTIC sore	6	5–7
Procalcitonin on day 1 (ng/mL)	1.17	0.2–6.1
Charlson comorbidity index (CCI)	4.5	2–7
Cardiovascular disease	23	(46.0%)
Cerebral vascular disease	33	(66.0%)
Chronic lung disease	21	(42.0%)
Chronic liver disease	18	(36.0%)
Diabetes Mellitus	19	(38.0%)
Chronic renal disease	19	(38.0%)
Malignancy	22	(44.0%)
Steroid for shock on day 1 (*n*, %)	42	84.0%
Mechanical ventilator days	9	6–17.3
ICU stays	12	8–18.5
Hospital stays	21.5	15.8–40
ICU mortality (*n*,%)	13	26.0%
Hospital mortality (*n*,%)	18	36.0%
Culture results (*n*,%)		
BAL culture (−)	29	58.0%
BAL culture (+)	21	42.0%
BAL (+) and EA (+)	13	26.0%
BAL (+) but EA (−)	8	16.0%
Antibiotics prescription on day 1		
Anti-*Pseudomonas*	39	78.0%
Anti-*Pseudomonas* + Anti-*MRSA*	3	6.0%
Non-*Pseudomonas*, Non-*MRSA* coverage	8	16.0%
Changes of antibiotics prescription on day 3 adjusted by BAL culture results (*n*, %)	9	18.0%
Rationale for changing prescription in antibiotics		
De-escalation due to negative of *Pseudomonas*	7	77.8%
*MRSA* or *Pseudomonas* positive from BAL	2	22.2%

Acute Physiology and Chronic Health Evaluation II: APACHE II; Sequential Organ Failure Assessment: SOFA; Modified Nutrition Risk in Critically Ill: mNUTRIC; Intensive Care Unit: ICU; Bronchoalveolar lavage: BAL; Endotracheal aspirate: EA; Methicillin-resistant Staphylococcus aureus: MRSA.

**Table 2:** Patient demographic characteristics, severity index, comorbidities, and antibiotic prescription pattern according to the BAL culture results (*n* = 50).

Characteristics	BAL Culture (−) (*n* = 29, 58%)	BAL Culture (+) (*n* = 21, 42%)	*p*
Age	75.3 (66–84.2)	75.8 (64.7–87.7)	0.549
Gender (*n*, Male %)	15 (51.7%)	18 (85.7%)	0.228 *
Body mass index	23.6 (20.3–26.9)	22.3 (18.6–25.0)	0.302
APACHE II	29.0 (24.5–34.5)	28.0 (24.5–33.0)	0.836
SOFA score	8.0 (6.0–12.0)	6.0 (4.5–10.0)	0.139
mNURTIC sore	6.0 (5.5–7.0)	6.0 (5.0–7.0)	0.474
Procalcitonin on day 1 (ng/mL)	1.0 (0.2–5.7)	1.2 (0.5–8.1)	0.541
Charlson comorbidity index (CCI)	5.0 (2.0–7.0)	3.0 (0.5–6.0)	0.281
Cardiovascular disease	16 (55.2%)	7 (33.3%)	0.214
Cerebral vascular disease	18 (62.1%)	15 (71.4%)	0.699
Chronic lung disease	12 (41.4%)	9 (42.9%)	1.000
Chronic liver disease	12 (41.4%)	6 (28.6%)	0.527
Diabetes Mellitus	10 (34.5%)	9 (42.9%)	0.759
Chronic renal disease	11 (37.9%)	8 (38.1%)	1.000
Malignancy	15 (51.7%)	7 (33.3%)	0.315
Steroid for shock on day 1 (*n*, %)	24 (82.8%)	18 (85.7%)	1.000
Mechanical ventilator days	11.0 (6.5–21.0)	8.0 (5.5–12.5)	0.110
ICU stays	15.0 (10.5–23.0)	10.0 (7.5–14.5)	0.061
Hospital stays	22.0 (15.5–37.0)	20.0 (15.5–41.5)	0.984
ICU mortality (*n*, %)	10 (34.5%)	3 (14.3%)	0.200
Hospital mortality (*n*, %)	14 (48.2%)	4 (19.0%)	0.383
Antibiotics prescription on day 1			0.801
Anti-*Pseudomonas*	21 (72.4%)	17 (81.0%)	
Anti-*Pseudomonas* + Anti-*MRSA*	2 (6.9%)	1 (4.8%)	
Non-*Pseudomonas*, Non-*MRSA*	5 (17.2%)	3 (14.3%)	
Changes of antibiotics on day 3 adjusted by BAL (*n*, %) ^a^	6 (20.7%)	3 (14.3%)	0.716
Rationale for changing in antibiotics			0.276
De-escalation due to *Pseudomonas* (−)	5 (83.3%)	2 (66.7%)	
*MRSA* or *Pseudomonas* (+) from BAL	1 (16.7%)	1 (33.3%)	

^a^ Fisher’s Exact Test; * *p* < 0.05.

## Data Availability

The data presented in this study are available on request from the corresponding author. The data are not publicly available due to the regulation of the Institutional Review Board of Taichung Veterans General Hospital in Taiwan.

## Supplementary Materials

The following supporting information can be downloaded at https://www.mdpi.com/article/10.3390/jcm11020327/s1: Supplementary Figure S1: (A) The correlation between microbial communities was the same in the EA samples; (B) The correlation between microbial communities was the same in the BAL samples.; (C) Distribution of rho correlations between microbial communities in EA and BAL samples.
