# Supplementary material for: Compared the Microbiota Profiles between Samples from Bronchoalveolar Lavage and Endotracheal Aspirates in Severe Pneumonia: A Real-World Experience
Source: J Clin Med. 2022 Jan 10;11(2):327. doi: 10.3390/jcm11020327 (PMC8778781; doi:10.3390/jcm11020327)
Supplement: Supplementary file 1 [file jcm-11-00327-s001.zip › jcm-1496353-supplementary.pdf]

Supplementary Material

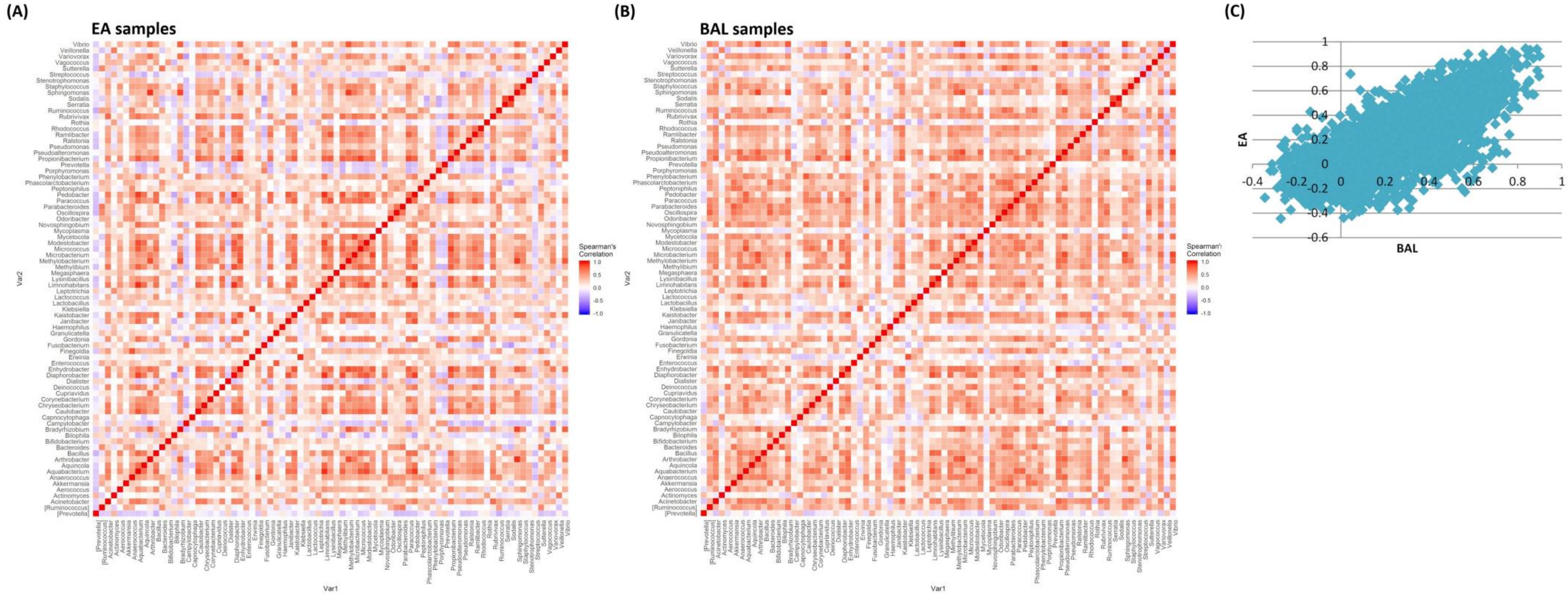

Figure S1. (**A**) The correlation between microbial communities was the same in the EA samples; (**B**) The correlation between microbial communities was the same in the BAL samples.; (**C**) Distribution of rho correlations between microbial communities in EA and BAL samples.
